# INTEGRATE-Circ and INTEGRATE-Vis: unbiased detection and visualization of fusion-derived circular RNA

**DOI:** 10.1093/bioinformatics/btad569

**Published:** 2023-09-14

**Authors:** Jace Webster, Hung Mai, Amy Ly, Christopher Maher

**Affiliations:** Department of Internal Medicine, Washington University School of Medicine, St. Louis, MO 63110, United States; Department of Internal Medicine, Washington University School of Medicine, St. Louis, MO 63110, United States; Department of Internal Medicine, Washington University School of Medicine, St. Louis, MO 63110, United States; Alvin J. Siteman Cancer Center, Washington University School of Medicine, St. Louis, MO 63110, United States; Department of Internal Medicine, Washington University School of Medicine, St. Louis, MO 63110, United States; Alvin J. Siteman Cancer Center, Washington University School of Medicine, St. Louis, MO 63110, United States; Department of Biomedical Engineering, Washington University School of Medicine, St. Louis, MO 63130, United States

## Abstract

**Motivation:**

Backsplicing of RNA results in circularized rather than linear transcripts, known as circular RNA (circRNA). A recently discovered and poorly understood subset of circRNAs that are composed of multiple genes, termed fusion-derived circular RNAs (fcircRNAs), represent a class of potential biomarkers shown to have oncogenic potential. Detection of fcircRNAs eludes existing analytical tools, making it difficult to more comprehensively assess their prevalence and function. Improved detection methods may lead to additional biological and clinical insights related to fcircRNAs.

**Results:**

We developed the first unbiased tool for detecting fcircRNAs (INTEGRATE-Circ) and visualizing fcircRNAs (INTEGRATE-Vis) from RNA-Seq data. We found that INTEGRATE-Circ was more sensitive, precise and accurate than other tools based on our analysis of simulated RNA-Seq data and our tool was able to outperform other tools in an analysis of public lymphoblast cell line data. Finally, we were able to validate *in vitro* three novel fcircRNAs detected by INTEGRATE-Circ in a well-characterized breast cancer cell line.

**Availability and implementation:**

Open source code for INTEGRATE-Circ and INTEGRATE-Vis is available at https://www.github.com/ChrisMaherLab/INTEGRATE-CIRC and https://www.github.com/ChrisMaherLab/INTEGRATE-Vis.

## 1 Introduction

Circular RNAs (circRNAs) occur when splicing mechanisms cause downstream exons to covalently bind to an upstream exon, referred to as a backsplice, resulting in a circular, rather than linear, transcript. Backsplicing events are thought to rely primarily on standard spliceosome machinery and are in part facilitated by complimentary sequences located within the introns that flank the donor and acceptor splice sites, although trans-acting factors are also involved (Chen and Yang 2015). CircRNAs have been shown to function through a variety of mechanisms, including direct regulation of transcription (Li *et al.* 2015), indirect transcriptional regulation through interactions with microRNAs (Hansen *et al.* 2013, Memczak *et al.* 2013) or RNA-binding proteins (Huang *et al.* 2020), and by encoding peptides (Othoum *et al.* 2020). As circRNAs are not susceptible to degradation by exonucleases due to their circular structure, they are thought to be more stable than linear transcripts (Wang and Liu 2022, Zhang *et al.* 2018).

Fusion-derived circRNAs (fcircRNAs) are circRNAs that are generated by backsplicing within a gene fusion transcript and represent a recently discovered (Guarnerio *et al.* 2016) and poorly understood subset of circRNAs (Visci *et al.* 2020). The gene fusion transcripts that form fcircRNAs are typically the result of genomic structural variation, such as translocations or deletions, that cause the 5′ end of a gene to become juxtaposed to the 3′ end of an independent gene. Such gene fusions are common in many cancers (Soda *et al.* 2007, Wang *et al.* 2017, Nickless *et al.* 2022) and have been identified as druggable targets (Drilon 2019, Braun *et al.* 2020). FcircRNAs can also have other sources, such as the backsplicing of read-through transcripts that contain multiple genes, although these have sometimes been referred to as read-through circRNAs (rt-circRNAs) (Vo *et al.* 2019, Azatyan and Zaphiropoulos 2022). For simplicity, we will refer to any circRNA that is composed of multiple independent genes as fcircRNAs.

While fcircRNAs remain poorly understood, recent studies have demonstrated that they are functional. For example, fcircRNAs from *BCR*::*ABL1*, *PML*::*RARα* and *MLL*::*AF9* fusions have each shown oncogenic potential in leukemia (Guarnerio *et al.* 2016, Tan *et al.* 2021) and fcircRNAs arising from *EML4*::*ALK* fusions were shown to promote cell migration and invasion in non-small cell lung cancer (Tan *et al.* 2018b). An additional 62 fcircRNAs have been reported within RNA-Seq data across a cohort of prostate cancer patients, but their potential functions were not investigated (Chen *et al.* 2019). Considering the stability of circular transcripts and the somatic nature of most gene fusions, it is perhaps no surprise that early attempts have already been made to determine if fcircRNAs can be leveraged as cancer biomarkers (Tan *et al.* 2018a).

Despite the oncogenic nature of some fcircRNAs and their potential as biomarkers, the study of fcircRNAs has been severely limited due to (1) the widespread use of Poly(A)-selection in RNA protocols which systematically removes circRNAs prior to sequencing and (2) a lack of software tools capable of detecting such events. As a result, most previously identified fcircRNAs were discovered through targeted sequencing of hypothetical backsplice junctions in gene fusions of interest (Guarnerio *et al.* 2016). We are aware of only three software tools developed for fcircRNA detection. The first published tool, Acfs (You and Conrad 2016), has systematic biases by algorithmically requiring fcircRNAs to be formed by fused genes originating from different chromosomes or from different strands of the same chromosome (removing the possibility of detecting an fcircRNA from a read-through transcript or from well-studied fusions like *TMPRSS2*::*ERG*). The second tool, Fcirc (Cai *et al.* 2020), accepts unaligned reads as input and then uses a built-in aligner to map reads against custom reference sequences generated based on a user-supplied list of potential gene fusions. Finally, CircFusion (Dal Molin *et al.* 2023) uses a nearly identical workflow as Fcirc, but uses STAR (Dobin *et al.* 2013) for performing read alignments. Interestingly, both Fcirc and CircFusion require *a priori* knowledge via an input list of potential gene fusions thereby preventing unbiased fcircRNA discovery. Notably, the gene fusion list provided by Fcirc contains twice as many fusions as the list provided by CircFusion, highlighting an immediate discrepancy in the potential candidates that could be detected between tools. To our knowledge, there are no automated methods that allow the unbiased discovery of fcircRNAs throughout the full genome.

To address the need for improved fcircRNA detection methods, we have developed INTEGRATE-Circ. INTEGRATE-Circ is an open-source software tool capable of integrating both RNA and whole-genome sequencing (WGS) data to perform unbiased detection of novel gene fusions and report the presence of splice variants in gene fusion transcripts, including backsplicing events. We assessed the performance of INTEGRATE-Circ using simulated data and then demonstrated its utility through the analysis of leukemia and breast cancer cell lines. Additionally, we have released an update to our previously published tool, INTEGRATE-Vis, making it the first software capable of automatically generating publication-ready visualizations of fcircRNAs.

## 2 Materials and methods

### 2.1 INTEGRATE-Circ software

INTEGRATE-Circ leverages an algorithm originally developed for our highly accurate fusion discovery software, INTEGRATE. The original INTEGRATE algorithm was designed to analyze RNA-Seq, and when available include WGS, paired-end reads to detect high confidence, novel gene fusion events. A comparison with eight gene fusion detection tools demonstrated that INTEGRATE was the most accurate method. As such, the methodology behind INTEGRATE serves as a strong starting point for developing tools that can detect junctions between fused genes.

A thorough explanation of the original INTEGRATE fusion detection algorithm is provided in the INTEGRATE publication (Zhang *et al.* 2016), but a brief overview is provided here to give context for the changes that are implemented in INTEGRATE-Circ. The original workflow involves the creation of a gene graph such that each node consists of a gene and each edge is based on discordantly mapped read pairs that may encompass a fusion junction between the two genes. Initial pruning of the graph is performed, primarily through the re-alignment of discordant read pairs. Potential spanning reads and previously unmapped reads are then mapped to remaining gene nodes and their “neighboring node(s)” in an attempt to identify spanning read support for putative fusions and reads that are aligned near each other are clustered together to identify potential fusion junctions. Fusion junctions that are supported by the mapped RNA-Seq spanning reads are then compared against WGS reads to allow for single-base pair resolution of the genomic breakpoints, if WGS data are provided.

**Figure 1.**
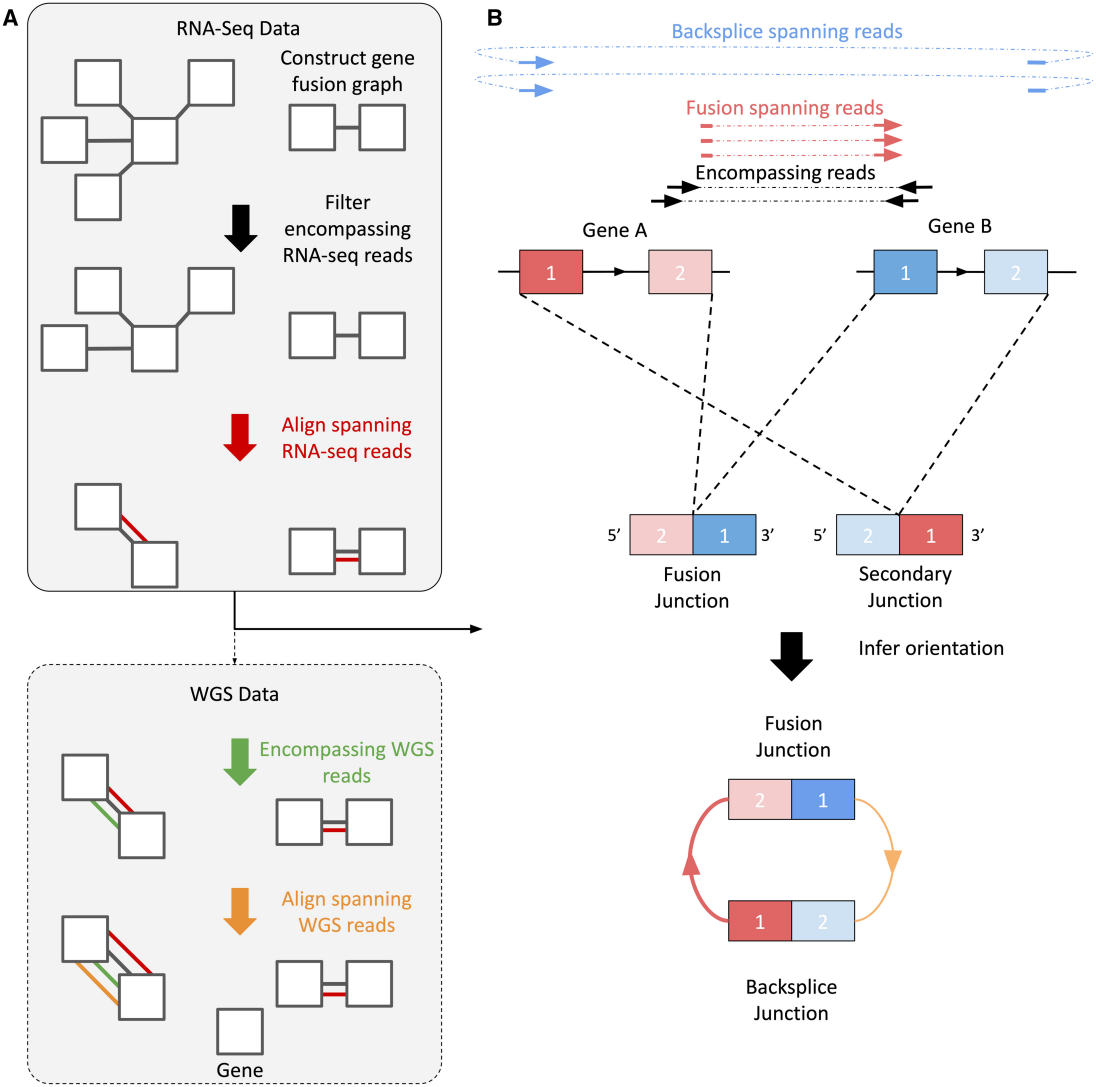
INTEGRATE-Circ workflow. (A) INTEGRATE-Circ beings by creating a gene graph of potential fusions based on RNA-Seq data and removing nodes from the graph based on encompassing and spanning read support. If provided, encompassing and spanning WGS reads are then examined for additional evidence for fusions. (B) Once gene fusion candidates have been identified, all RNA reads that span both genes are clustered together based on region to identify the locations of gene fusions. The junction with the most support is identified as the fusion junction and all other junctions are then evaluated based on their orientation and positioning with respect to the fusion junction.

INTEGRATE-Circ builds upon the INTEGRATE framework by using the location and orientation of detected junctions to infer the existence of unique isoforms generated by alternative splicing or backsplicing mechanisms. A general overview of this workflow is depicted in Fig. 1. After identifying potential gene fusions, clusters of junction-spanning RNA-Seq reads are re-evaluated. For each potential fusion, each cluster of spanning RNA-Seq reads are compared with each other to determine which cluster has the highest read support, with the most well-supported junction being considered the primary fusion. The primary fusion is expected to correspond to the true genomic fusion junction and should be supported by WGS data, if available. All other spanning read clusters are then evaluated with respect to the primary fusion junction. Since secondary junctions are thought to result from alternative splicing of transcripts and are not expected to be a direct indication of genomic rearrangements, secondary junctions are not expected to have WGS support. A simplified schematic demonstrating how spanning read clusters are annotated based on their relative orientation to the primary junction is depicted in Supplementary Fig. S1, although a much broader variety of potential secondary junction orientations, including those that do not match with canonical exon boundaries, are possible. INTEGRATE-Circ applies an extended version of the logic described in the schematic to all identified gene junctions. Where possible, junctions are compared based on canonical exon boundaries to aid in identifying reciprocal gene fusions. In cases where identified junctions are not located at annotated exon boundaries, relative locations and orientations are evaluated based on genomic base pair position for annotation purposes. By combining insights from RNA-Seq and WGS, INTEGRATE-Circ is designed to sensitively detect gene fusion junctions and be able to differentiate between genomic rearrangements and alternatively spliced transcripts, including backsplices.

All identified junctions from linear and circular transcripts (including read-throughs) are reported by INTEGRATE-Circ using a number of standardized formats, including bedpe, vcf, and generic tsv formats with accompanying annotation information (including gene names, total RNA-Seq/WGS read support, a list of supporting reads, and whether the junction uses canonical exon boundaries). Although bedpe and vcf files are commonly used for annotating standard fusion breakpoints, no standardized file format exists to specifically describe fcircRNAs. Therefore, INTEGRATE-Circ reports fcircRNAs using a modified bedpe file, described in the README file of the project GitHub page. The modified bedpe format is consistent with the output file generated by Fcirc to help ensure consistency with other downstream applications in the future. This file format is accepted by INTEGRATE-Vis for fcircRNA visualization. Additional details can be found at https://github.com/ChrisMaherLab/INTEGRATE-CIRC and in the Supplementary Materials.

### 2.2 INTEGRATE-Vis software

The fcircRNA visualization workflow within INTEGRATE-Vis consists of two primary steps: annotation and visualization. The annotation step uses a user provided, standard GTF file to determine the exon boundaries of exons located immediately around the reported fusion and backsplice junctions. If a junction does not match canonical exon boundaries, the nearest upstream (for 5′ end of junctions) or downstream (for 3′ end of junctions) exon boundary is selected for visualization purposes. Additionally, genomic cytoband information for the chromosome(s) involved in the fcircRNA is extracted from the user-provided ideogram file in order to put the genomic location of the fcircRNA into context.

The second step in the workflow is the creation of the visualization using the annotation information generated during the previous step. The genomic locations of fusion genes are presented based on cytoband location and the resulting fusion gene transcript is presented. The presented fusion transcript contains a minimal number of exons (max of 3 per gene), but does not necessarily represent the full transcript length, nor are the exons presented to scale. The fcircRNA is then presented in relation to the fusion transcript. Optionally, the user may provide a bam file from which INTEGRATE-Vis will attempt to identify the number of spanning reads that support both reported junctions. As both INTEGRATE-Circ and Fcirc perform their own custom secondary alignment steps, it is possible that the read support values calculated by INTEGRATE-Vis will differ from those reported by INTEGRATE-Circ and/or Fcirc. Additional details can be found at https://github.com/ChrisMaherLab/INTEGRATE-Vis.

## 3 Results

### 3.1 *In silico* simulation

**Figure 2.**
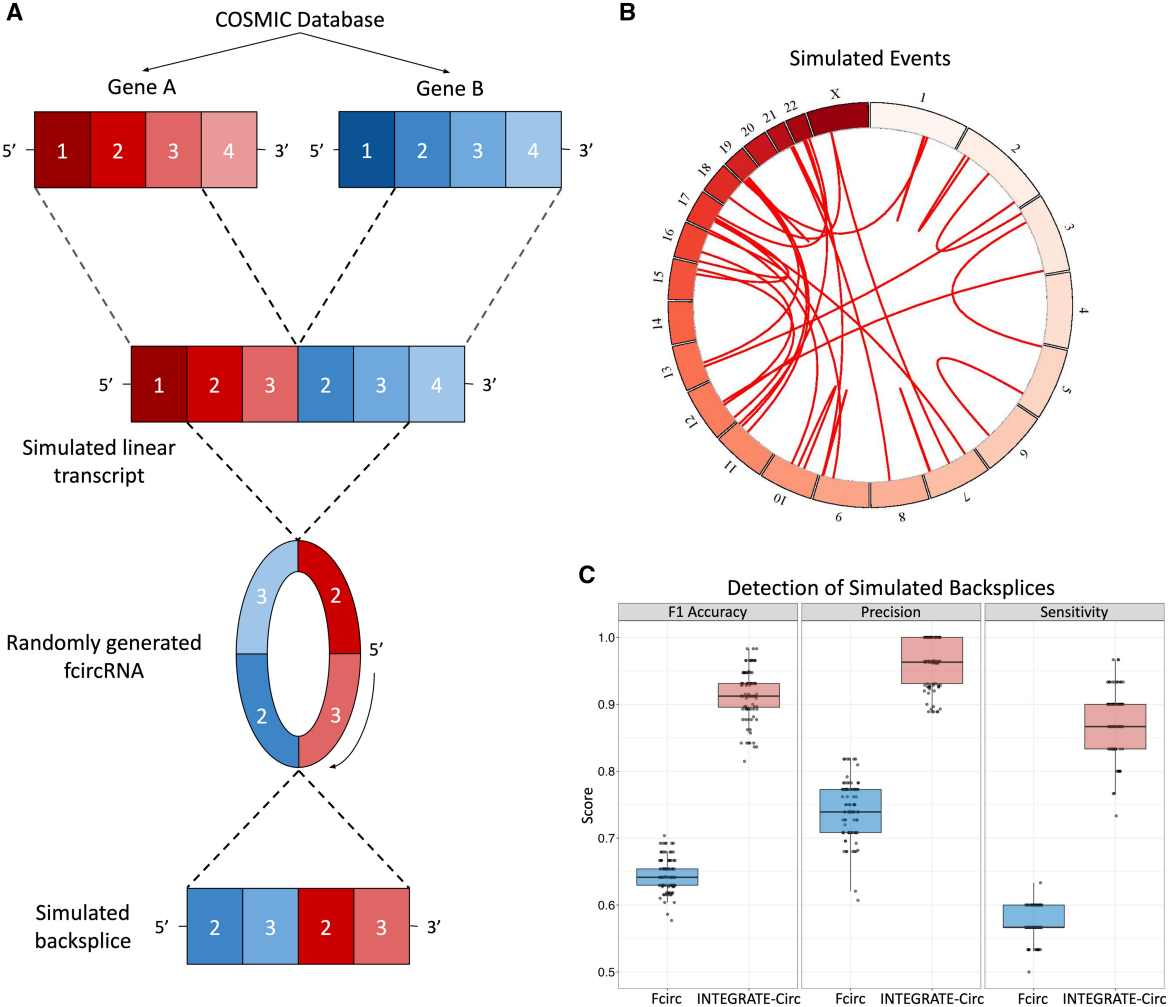
Benchmarking results based on *in silico* simulation. (A) Schematic depicting the creation of simulated linear fusion and backsplice transcripts. Recurrent gene fusions were identified from the COSMIC database and theoretical backsplice junctions were then randomly introduced to the selected fusions. Linear fusion transcripts and linearized versions of the regions that spanned the simulated backsplices were used to simulate RNA-Seq reads. (B) Circos plot representing all simulated events. (C) F1 accuracy, precision, and sensitivity scores after analysis of the simulated fcircRNAs by both tools across 100 iterations. Acfs is not shown as no fcircRNA calls were made.

To perform an initial comparison between the identified fcircRNA detection tools, a simulated dataset containing 30 linear fusion transcripts was generated based on the most frequent gene fusions reported in the Gene Fusion Curation portion of COSMIC v96 database (Tate *et al.* 2019). Randomly generated backsplice junctions were then created for each of the fusion transcripts based on the exons present in the reported fusion transcript. RNA-sequencing reads for the fusion and backsplice junctions were simulated 100 separate times. An overview of this workflow and the resulting simulated inter- and intra-chromosomal events can be found in Fig. 2A and B and Supplementary Table S1. Additional details regarding the simulation of the data and the resulting transcripts can be found in the Supplementary Methods and Additional File S1.

For benchmarking purposes, INTEGRATE-Circ, Fcirc, Acfs, and CircFusion were applied to the simulated data with default settings (except Acfs, which was given the “Search_trans_splicing yes” parameter to support fcircRNA detection). Since Fcirc and CircFusion both require a list of potential gene fusions, the provided gene fusion lists (downloaded from GitHub for each tool on March 10, 2022 and Feburary 7, 2023, respectively) were used as input (305 fusions for CircFusion and 773 fusions for Fcirc). CircFusion failed to run with default settings with exit warnings suggesting that the 305 gene fusion list was too large. This failure, combined with the fact that CircFusion accepts the expected transcript IDs, fusion breakpoints, and backsplice junctions of potential fcircRNAs, suggests that CircFusion may be better optimized for validation of specific, previously identified events and led us to exclude CircFusion from the remaining benchmarking analyses. Acfs did not report any fcircRNAs in our simulated data (and reported no errors at runtime). Although the tool can be applied to paired-end sequencing data, it was primarily designed for single-read data and the authors have indicated that the tool may have lower sensitivity when using paired-end reads (all our data are paired-end). Acfs also failed to detect any fcircRNAs in sequencing data from tissue samples in the original publication, further corroborating the poor sensitivity we observed. Sensitivity, precision, and F1 accuracy scores for results from INTEGRATE-Circ and Fcirc were then calculated for each of the 100 simulation iterations. We found that when comparing fcircRNA detection between INTEGRATE-Circ and Fcirc, INTEGRATE-Circ was superior in terms of sensitivity (mean: 87.3% ± 4% versus 57.1% ± 2%), precision (mean: 96.1% ± 3% versus 74.2% ± 4%), and F1 accuracy (mean: 91.5% ± 4% versus 64.5% ± 2%) (Fig. 2C). Notably, if Acfs had consistently achieved the maximum sensitivity that its algorithm would allow (it systematically excludes four of the simulated backsplices because their contributing genes originated on the same strand of the same chromosomes), the maximum possible sensitivity of the tool in this simulation would be 86.6%, meaning that it could not have outperformed INTEGRATE-Circ’s average sensitivity.

### 3.2 Application to public K562 cell line data

Next, we applied INTEGRATE-Circ, Acfs, and Fcirc to the K562 lymphoblast cell line (SRA Accession: SRR8587462) which contains four validated linear fusion transcripts, three of which have published support for the presence of fcircRNAs in either K562 (Tan *et al.* 2021) or in a different context (Vo *et al.* 2019, Azatyan and Zaphiropoulos 2022). A summary of the results regarding the four previously validated fusion transcripts are shown in Table 1. For previously published junctions, we required one or more reads. For novel junctions we required two or more independent reads. We found that Fcirc detected only one of the published linear gene fusions and no corresponding fcircRNAs while INTEGRATE-Circ detected all four linear fusions and reported fcircRNAs in three of the four fusions. Of the three fcircRNAs called by INTEGRATE-Circ, two have been previously reported [circ*PRKAA1*(5,6,7,8,9,10)::*TTC33*(1,2) (Vo *et al.* 2019) and circ*KANSL1*(3)::*ARL17A*(3) (Azatyan and Zaphiropoulos 2022)] while one [circ*NUP214*(25,26,27,28,29)::*XKR3*(2,3)] was novel. Unfortunately, neither tool was able to detect the circ*BCR*(13,14)::*ABL1*(2,3) fcircRNA that was previously reported in this cell line (Tan *et al.* 2021), however this result is consistent with a previous attempt to detect fcircRNAs using this same public sequencing data which also failed to detect the circ*BCR*(13,14)*::ABL1*(2,3) isoform (Vo *et al.* 2019).

**Table 1.** Detection of linear and backsplice junctions in K562 cell line.

	Read support			
	Fusion junction		Backsplice junction	
Fusion	INTEGRATE-Circ	Fcirc	INTEGRATE-Circ	Fcirc
*BCR::ABL1*	960	1146	–	–
*PRKAA1::TTC33*	12	–	1	–
*KANSL1::ARL17A*	35	–	9	–
*NUP214::XKR3*	356	–	3	–

Supporting reads for previously reported linear fusion transcripts in K562 and any fcircRNAs that may derive from those transcripts. Missing values indicate that no junction was reported. Acfs has been excluded, as it cannot report linear fusions and because it reported no fcircRNAs.

### 3.3 Application to HCC1395 cell line

For a final evaluation, we applied INTEGRATE-Circ, Acfs, and Fcirc to the breast cancer cell line HCC1395. This cell line was chosen because it has significantly more validated fusions than K562 but has not previously been evaluated for the presence of fcircRNAs.

**Figure 3.**
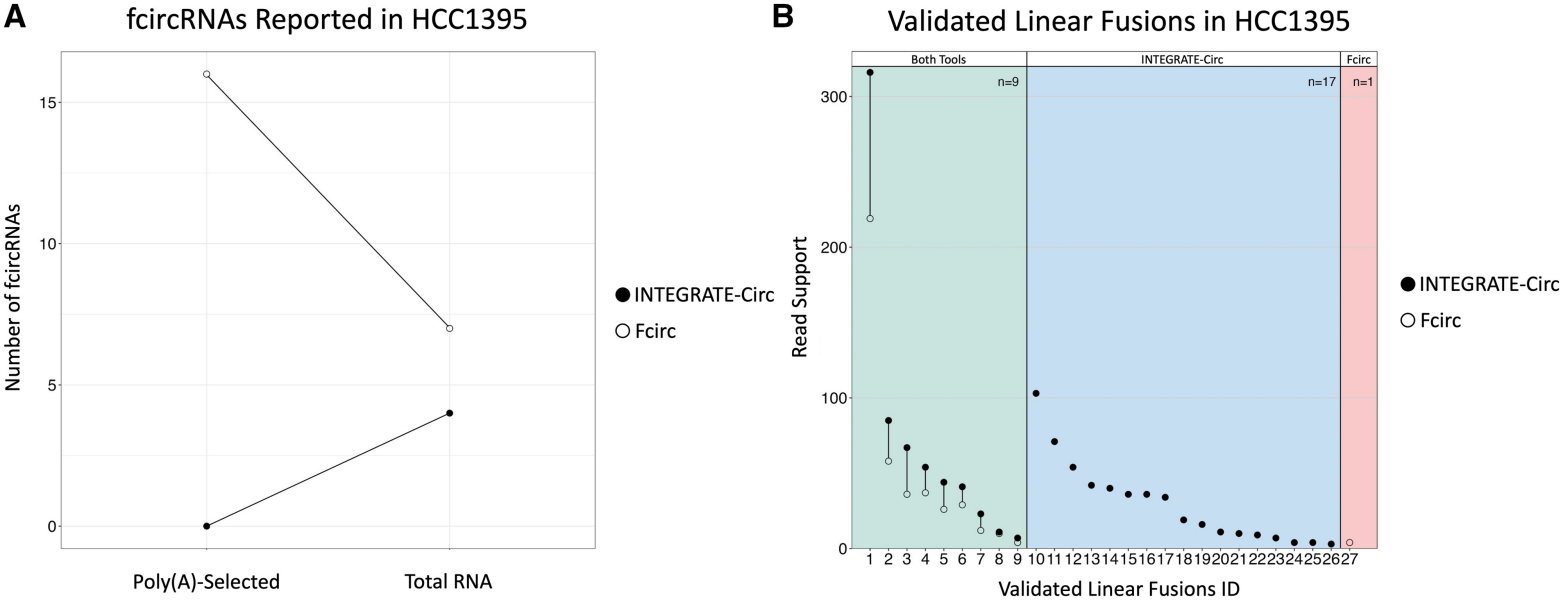
Analysis of HCC1395 cell line data. (A) Number of fcircRNAs reported by INTEGRATE-Circ and Fcirc when applied to Poly(A)-selected and total RNA sequencing data. (B) Reported read support for previously validated HCC1395 linear fusions. Acfs has been excluded, as it cannot report linear fusions and because it reported no fcircRNAs.

To ensure that each tool was working as intended, both Poly(A)-selected and total RNA sequencing data were analyzed. We expect that no fcircRNAs would be found in the Poly(A)-selected data due to the removal of circular transcripts during the Poly(A) enrichment. As anticipated, INTEGRATE-Circ only reported fcircRNAs in the total RNA data (Fig. 3A). In contrast, Fcirc unexpectedly nominated 16 fcircRNAs in the Poly(A)-selected data, nearly 3× more fcircRNAs than it reported in the total RNA data. None of the fcircRNAs called by Fcirc in the total RNA data were reported in the Poly(A)-selected data or vice versa. We focused our remaining analysis only on the total RNA data results since fcircRNAs observed in the Poly(A)-selected data were thought to be potential noise and are suggestive of Fcirc having a potentially high false positive rate.

#### 3.3.1 Detection of validated fusions

As all fcircRNAs must, by definition, be a subset of the detected fusion transcripts, we next compared INTEGRATE-Circ and Fcirc linear fusion calls made using the total RNA data against a published list of validated fusions in HCC1395 (Zhang *et al.* 2016), requiring more than two supporting independent reads. In the nine validated fusions that were reported by both tools, we found that INTEGRATE-Circ reported greater read support in 100% of the fusions (Fig. 3B). Additionally, 17 previously reported fusions were found by INTEGRATE-Circ alone while only 1 published event was found solely by Fcirc. Two additional validated gene fusions called by Fcirc failed to meet our filtering criteria (Supplementary Fig. S2A–C) and were missed by INTEGRATE-Circ because the reads were either not mapped to the gene of interest and/or no encompassing reads were detected (which is required by INTEGRATE-Circ).

#### 3.3.2 *In vitro* validation of novel fcircRNAs

**Figure 4.**
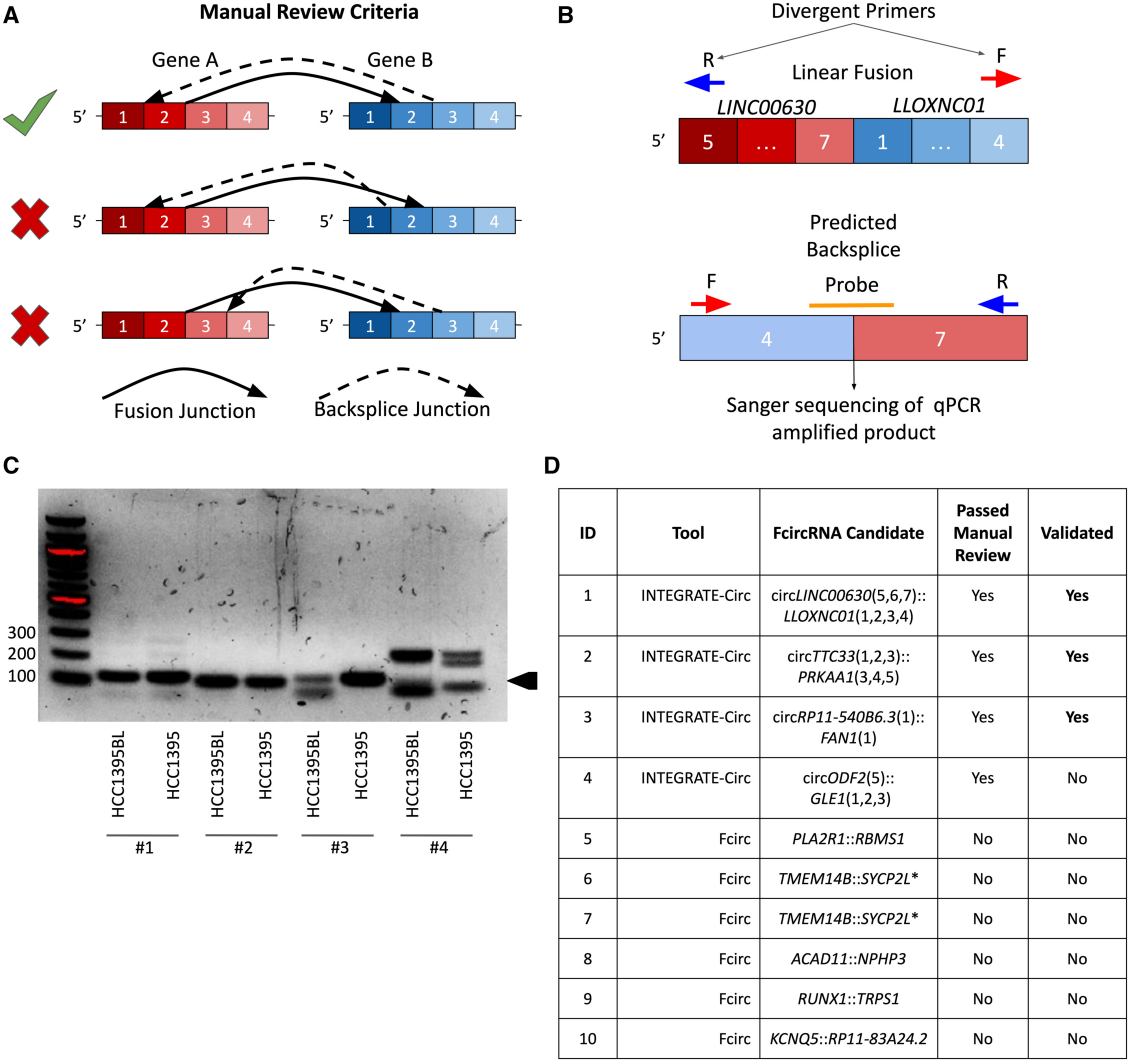
Validation of HCC1395 fcircRNAs. (A) Manual review process for fcircRNAs. While the top example represents the expected relative locations of fusions and backsplice junctions, the other schematics represent scenarios where the backsplice donor would not be present in the fusion transcript (middle example) or the backsplice acceptor would not be present in the fusion transcript (bottom example). Reported fcircRNAs that follow the middle or bottom examples are physically impossible as an fcircRNA must be a subset of the sequence present in the fusion transcript and were therefore excluded from PrimeTime Probe qPCR validation. (B) Design of divergent forward and reverse primers that face away from the fusion junction and placement of PrimeTime Probes to span the reported backsplice junction. The design for circLINC00630(5,6,7)::LLOXNC01(1,2,3,4) is shown but an identical procedure was used for each candidate that was evaluated. (C) Gel of the amplified PrimeTime Probe qPCR products for both the HCC1395 cancer cell line and the matched normal tissue HCC1395BL. Bands of the expected size were excised and sent for Sanger sequencing. Black arrow indicates expected size. (D) All reported fcircRNA candidates. A validation status of “Yes” denotes that the Sanger sequencing of PrimeTime Probe qPCR pdocuts matched the expected backsplice junction sequence. The * indicates that multiple fcircRNA isoforms were reported to result from the same fusion transcript. Candidates that failed manual review are not shown with standard notation because their orientations (as in panel A) meant that they could not be accurately described using this nomenclature.

Finally, we attempted to validate predicted fcircRNAs using PrimeTime Probe reverse transcription quantitative PCR (PrimeTime Probe qPCR) amplification of putative backsplices (Supplementary Methods). Divergent primers for all fcircRNA candidates that passed manual review (Fig. 4A and Supplementary Methods) from either tool were designed using the strategy depicted in Fig. 4B, as has been described previously for both circRNA (Panda and Gorospe 2018) and fcircRNA (Guarnerio *et al.* 2016) validation. We also performed the PrimeTime Probe qPCR assay on the HCC1395 B Lymphocyte (HCC1395BL) cell line, which serves as a matched normal control cell line. PrimeTime Probe qPCR amplified products were run on a gel (Fig. 4C) and purified products from the HCC1395 cell line were excised from the gel and Sanger sequenced, confirming the presence of the circ*TTC33*(1,2,3)::*PRKKA1*(3,4,5), circ*LINC00630*(5,6,7)*::LLOXNC01-237H1.2*(1,2,3,4), and circ*RP11-540B6.3*(1)*::FAN1*(1) fcircRNAs reported by INTEGRATE-Circ in HCC1395 (Fig. 4D). Notably, PrimeTime Probe qPCR products consistent with the size of circ*TTC33*(1,2,3)*::PRKKA1*(3,4,5) and circ*LINC00630*(5,6,7)*::LLOXNC01-237H1.2*(1,2,3,4) fcircRNAs were detected in the HCC1395BL cell line as well as the cancer cell line (Fig. 4C and Supplementary Fig. S3). As these fcircRNAs appear to be derived from read-through transcripts and are not the result of somatic structural variation, it is perhaps unsurprising that evidence for them was found in both cell lines, rather than only in the cancer cell line. We were unable to confirm the presence of any of the fcircRNAs reported by Fcirc. Similarly, as each validated fcircRNA was composed of genes from the same strands of the same chromosomes, the Acfs algorithm would not have been able to detect any of the validated fcircRNAs.

### 3.4 Updates to INTEGRATE-Vis visualization tool

**Figure 5.**
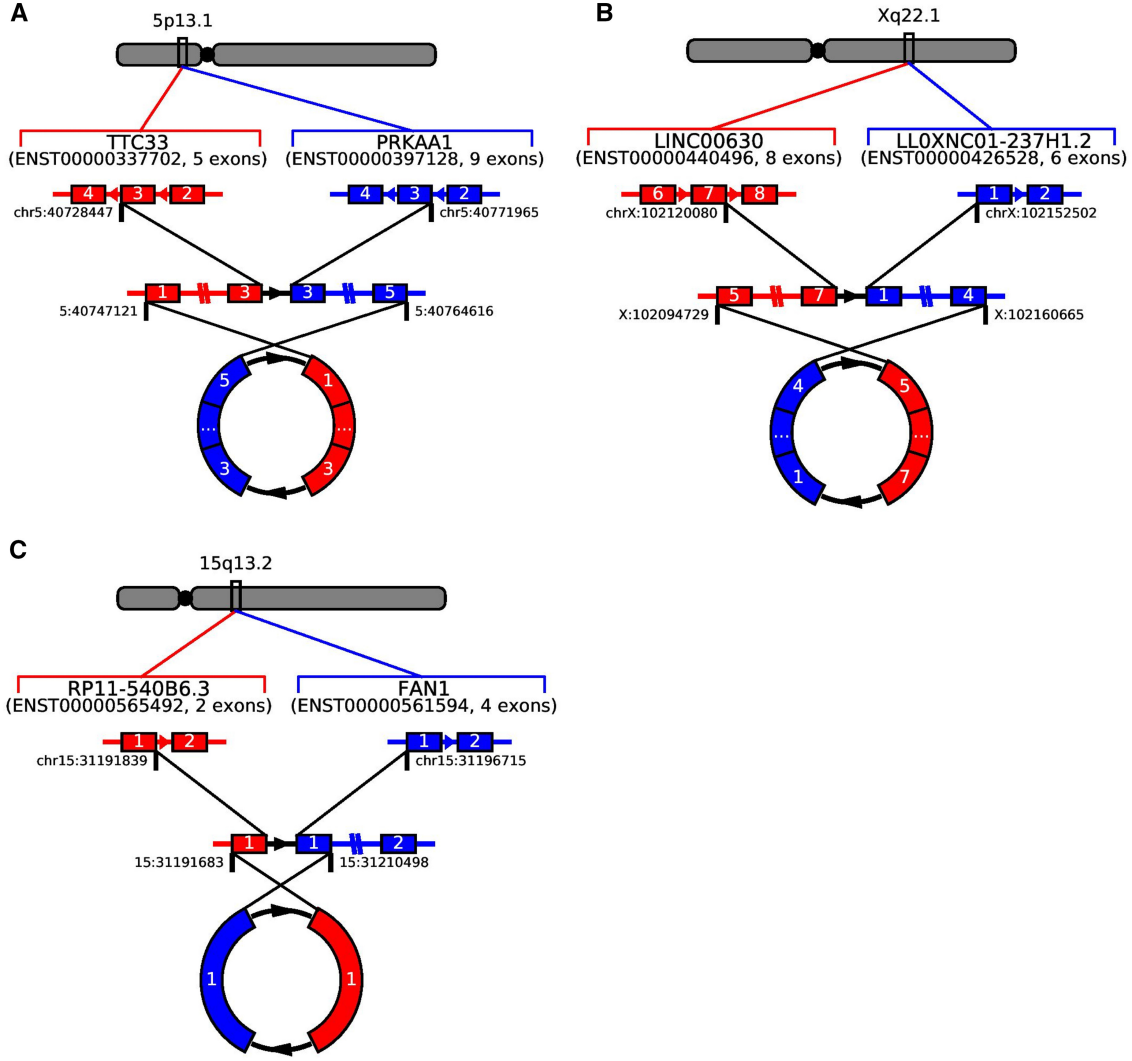
Validated HCC1395 fcircRNAs visualized with INTEGRATE-Vis. Default output from INTEGRATE-Vis depicting the validated (A) circTTC33(1,2,3)::PRKAA1(3,4,5), (B) circLINC00630(5,6,7)::LLOXNC01-237H1.2(1,2,3,4), and (C) circRP11-540B6.3(1)::FAN1(1) fcircRNAs.

Currently there are no publicly available tools for visualizing fcircRNAs. Most studies have relied on the manual creation of schematics to convey their findings, which can be time-consuming, leads to highly variable figure quality between studies, and can cause confusion when trying to accurately depict complex fcircRNA isoforms which, until recently, lacked a formalized nomenclature (Chen *et al.* 2023). To improve the dissemination of information in this relatively new field, we implemented an updated version of INTEGRATE-Vis (v1.1.0). In addition to the visualizations of linear fusion transcripts which were supported by earlier versions of INTEGRATE-Vis (Zhang *et al.* 2017), the tool now supports the visualization of detected fcircRNAs and is compatible with both INTEGRATE-Circ and Fcirc output files. Example outputs using default settings are shown in Fig. 5A–C, which depict the three novel, validated HCC1395 fcircRNAs identified by INTEGRATE-Circ.

## 4 Discussion

Here, we present both the novel tool, INTEGRATE-Circ, and v1.1.0 of INTEGRATE-Vis. Together, these open-source software tools allow for unbiased detection and visualization of novel fcircRNAs. Through the use of (1) simulated data, (2) publicly available cell line data, and (3) experimental validation in a paired breast cancer and normal cell line, we have demonstrated the ability of INTEGRATE-Circ to accurately identify linear fusion transcripts and fcircRNAs using short-read, paired-end sequencing data in an unbiased fashion.

One potential limitation of the INTEGRATE-Circ algorithm is that all annotations assume that the junction with the most spanning read support is the true fusion junction. This assumption may be false in situations where an alternative splice variant of a fusion transcript is more abundant than the transcript that represents the full genomic fusion. The inclusion of WGS data in the INTEGRATE-Circ algorithm is meant to minimize the likelihood of incorrectly designating an alternatively spliced junction as the primary junction, as the WGS reads should only support the true genomic fusion. Although users can run INTEGRATE-Circ without WGS data, including this information is likely to improve performance when trying to avoid such scenarios.

While fcircRNAs are composed of sequences from different genes, there are multiple ways for disparate gene sequences to become part of the same transcript, such as gene fusions and read-throughs. Each of the isoforms validated in the HCC1395 cell line in this study were the result of read-through transcripts, sometimes referred to as rt-circRNAs instead of fcircRNAs. Some events fit poorly into any current characterization, such as the circ*KANSL1*(3)::*ARL17A*(3) transcript identified by INTEGRATE-Circ in the K562 cell line and previously reported in a medulloblastoma patient and other cell lines (Vo *et al.* 2019, Azatyan and Zaphiropoulos 2022). *ARL17A* is immediately upstream of the adjacent *KANSL1* on chromosome 17, but their positions are inverted as *KANSL1* becomes the 5′ gene partner of the *KANSL1*::*ARL17A* fusion transcript that later gives rise to the associated circRNA (Azatyan and Zaphiropoulos 2022). The resulting circRNA is therefore not a typical read-through event, nor does it necessarily involve a genomic alteration. Indeed, *KANSL1*::*ARL17A* circularized transcripts have been referred to as both fcircRNAs (Azatyan and Zaphiropoulos 2022) and as rt-circRNAs (Vo *et al.* 2019) in published literature. In either case, the capability of INTEGRATE-Circ to detect circRNAs resulting from both read-throughs and larger intra-/inter-chromosomal fusions, as evidenced by our analysis of cell line data and a variety of simulated fusion events, is indicative of the broad utility of our unbiased approach.

As demonstrated by the performance of INTEGRATE-Circ in both breast cancer and leukemia cell lines, this approach has broad applicability independent of the cancer type. Indeed, as seen in our analysis of the healthy normal HCC1395BL cell line, fcircRNAs caused by read-throughs can be present even in healthy normal tissue. It is possible that fcircRNAs are more prevalent in diseases where structural variation is a common feature, but prior limitations have prevented comprehensive studies. Similarly, it is possible that their prevalence increases later in disease development due to the accumulation of somatic mutations. By providing improved detection and visualization methods, we hope that future work will be able to address such questions.

In summary, we have demonstrated that the novel software tool, INTEGRATE-Circ, can sensitively and accurately identify both linear fusion transcripts and fcircRNAs with single-base pair resolution, in an unbiased manner, across a variety of datasets. Additionally, the companion tool INTEGRATE-Vis is the first to provide automated visualization of fcircRNAs. We anticipate that the combined use of these tools will facilitate a wide variety of future studies to better understand the basic and clinical significance of fcircRNAs.

## Supplementary Material

btad569_Supplementary_Data

## Data Availability

The K562 and Poly(A)-selected HCC1395 datasets supporting the conclusions of this article are available in the Sequence Read Archive under accession numbers SRR8587462 and SRR892423, respectively. The simulated data supporting the conclusions of this article are included within the article and additional files. The total RNA HCC1395 data supporting the conclusions of this article are available in the Sequence Read Archive under PRJNA9726981. All software developed for this study is freely available at https://www.github.com/ChrisMaherLab/INTEGRATE-Vis and https://www.github.com/ChrisMaherLab/INTEGRATE-CIRC.
