# Supplementary material for: INTEGRATE-Circ and INTEGRATE-Vis: unbiased detection and visualization of fusion-derived circular RNA
Source: Bioinformatics. 2023 Sep 14;39(9):btad569. doi: 10.1093/bioinformatics/btad569 (PMC10516643; doi:10.1093/bioinformatics/btad569)
Supplement: btad569_Supplementary_Data [file btad569_supplementary_data.zip › SupplementaryMaterials.docx]

INTEGRATE-Circ and INTEGRATE-Vis: Unbiased Detection and Visualization of Fusion-Derived Circular RNA

Jace Webster, Hung Mai, Amy Ly, Christopher Maher

**Supplementary Materials**

**SUPPLEMENTARY METHODS**

**INTEGRATE-Circ software: Additional implementation details**

All benchmarking of INTEGRATE-Circ was performed on a big memory blade with 32 Intel Xeon CPU E5-2640s with 400G of memory. On our largest dataset (HCC1395 RNA-Seq data, ~200 million paired-end reads), the run time for INTEGRATE-Circ on the big memory blade was approximately 1.5 hours. We do note however that while all formal analysis was performed on a blade with 400G of memory, peak resident set size (RSS) was often significantly lower than the 400G available. For example, when analyzing our *in silico* simulated data, we recorded a peak RSS of approximately 5G.

INTEGRATE-Circ requires paired-end sequencing data to identify encompassing reads when creating the initial gene graph (Figure 1) and therefore cannot accept single-end reads. Required sequencing depth varies based on how highly expressed a given fcircRNA may be. In our analysis of HCC1395, a total of ~200 million paired-end reads were generated and all validated fcircRNAs had <5 supporting RNA-Seq reads. It is possible that deeper sequencing may be required for less abundant isoforms and to reduce the likelihood of false positives.

In order to determine optimal read length, we performed an *in silico* simulation identical to the one described in the main manuscript and below in the ‘Simulated data generation’ section, except that 100 iterations were performed with reads of variable length in ranges commonly used for short-read sequencing (2x50bp, 2x75bp, 2x100bp, 2x125bp and 2x150bp). The results indicate that optimal performance is achieved when using 2x75bp reads (Supplementary Figure 4). This is consistent with Illumina’s current official recommendation of using 2x75bp reads for transcriptome analysis (https://knowledge.illumina.com/library-preparation/rna-library-prep/library-preparation-rna-library-prep-reference_material-list/000001243).

**Simulated data generation**

The Gene Fusion Curation portion of the COSMIC v96 database(Tate *et al.*, 2019) was used to identify 30 recurrent gene fusions for use as a basis for the *in silico* simulation. Fusions were first ranked by the number of mutated samples and then the single most common isoform for each fusion was selected for simulation. No gene was permitted to appear in more than one selected fusion, meaning that some fusions were skipped because promiscuous genes were listed as frequently having multiple gene partners (for example, the *SS18*::*SSX1* and *SS18*::*SSX2* fusions were both highly recurrent, but only the *SS18*::*SSX1* fusion was selected). The positions of each individual exon present in the selected fusions were then identified using Ensembl’s hg19 annotation and individual exon sequences were then isolated using *bedtools getfasta -name -s -fi <hg19.fa> -bed <all_exons.bed> > exons.fa*. Backsplices with randomly generated junctions (using canonical exon boundaries) were then designed for each of the 30 fusions. All linear fusion isoforms selected from COSMIC and their corresponding fcircRNAs were then assembled by grouping together the necessary exons from the *exons.fa* file to form multi-exon transcripts.

Next, RNA-Seq reads were generated using the simReads() function from the Rsubread R library (R version 4.0.0, Rsubread version 2.4.3) (Liao *et al.*, 2019). The initial random seed was set to 42 and then reads were simulated over 100 iterations using the following parameters: *library.size = 100000, read.length = 100, paired.end = True, simulate.sequencing.error = True*. All transcripts used as input to the simReads() function are provided in Additional File 1. No WGS data was simulated.

**K562 data**

K562 cell line sequencing data was downloaded from the Sequence Read Archive using accession number SRR8587462. No WGS data was used for analysis. Fastq files were supplied directly to Fcirc with default settings. For INTEGRATE-Circ, reads were aligned as described below and then analyzed with default settings. For Acfs, reads were prepared by following the paired-end instructions on the project’s GitHub page.

**HCC1395 sequencing data**

Poly(A)-selected HCC1395 sequencing data was downloaded from the public Sequence Read Archive, accession number SRR892423. For Total RNA sequencing of HCC1395, a total RNA input of 1μg was used to generate the RNA-seq library using the New England BioLabs NEBNext Ultra II Directional RNA Library Prep for Illumina kit with rRNA Depletion module and NEBNext Multiplex Oligos for Illumina (Unique Dual Index UMI Adaptors RNA Set 1) per manufacturer’s protocol. Paired-end sequencing was performed on the NovaSeq platform.

**Manual review of fcircRNA calls in HCC1395**

Manual review was performed based on the fusion and backsplice junctions reported in the final output files of INTEGRATE-Circ and Fcirc to ensure that the junctions were in an orientation that was capable of forming an fcircRNA. An example of some of the patterns observed are presented in Figure 4A. As the orientation of the original genes and the junctions are known, it is possible to predict the potential outcomes of any combination of junctions. The top example in Figure 4A depicts the expected pattern during the manual review and describes an fcircRNA that would contain only the 2^nd^ exon of Gene A and the 3^rd^ exon of Gene B. The middle example from Figure 4A depicts a fusion junction connecting Gene A exon 2 to Gene B exon 2, but suggests that Gene B exon 1 is a backsplice donor. As Gene B exon 1 is not contained in the fusion transcript, it is not possible for Gene B exon 1 to be involved in the backsplice junction. Similarly, the final example in Figure 4A depicts a backsplice acceptor (Gene A exon 4) that would not be present in the linear fusion transcript. Scenarios such as these were excluded from PrimeTime qPCR validation as they were considered impossible and representative of a software error.

**Validation of fcircRNA calls in HCC1395**

Potential candidates were precisely validated using PrimeTime qPCR Probe Assays (Integrated DNA Technologies) with forward and reverse primer sets covering approximately 50nt each side of the backsplice junctions, and qPCR probes that specifically spanned the backsplice junctions themselves. Amplification was performed using the manufacturers recommended protocols. Amplified PrimeTime Probe qPCR products were purified using DNA Clean & Concentrator-5 (ZYMO RESEARCH) and purified DNA was analyzed with Sanger sequencing. Output from Sanger sequencing was compared to predicted backsplice junction sequences to assess the presence of the fcircRNA.

Selection and excision of DNA from the gel was done based on an expected size of approximately 100bp for each fcircRNA (based on the distance of primers from backsplice junctions, Fig 4B and 4C). Non-specific bands were thought to be caused by non-specific binding to different transcripts, in part due to non-ideal PCR conditions. For example, due to having a large number of primers being used during the PCR reactions, the annealing temperature was sub-optimal for a small number of primers. Similarly, due to the low expected abundance of fcircRNA, a relatively large number of PCR cycles were performed (45 cycles), which may also introduce non-specific binding.

**Alignment of all sequencing data**

Fcirc performs its own sequence alignment and accepts unaligned fastq files as input. Therefore, all reads from the *in silico* simulation, public K562 data and HCC1395 sequencing were provided to Fcirc in an unaligned format and were then aligned to hg19 by Fcirc.

In the original INTEGRATE publication (Zhang *et al.*, 2016), it was shown that performance can vary slightly based on the aligner used and that optimal performance was achieved by using GSNAP for initial alignment. For this reason, all reads analyzed by INTEGRATE-Circ were aligned by GSNAP (version 2021-03-08)(Wu and Nacu, 2010) using the following parameters: *-d hg19, --novelsplicing=1, --read-group-platform=Illumina, --extend-soft-clips*. Aligned reads were then sorted using *samtools sort* (v1.7)(Danecek *et al.*, 2021) and processed by INTEGRATE-Circ using default parameters. Although INTEGRATE-Circ accepts WGS data, no WGS data was used for benchmarking purposes in order to provide a fair comparison between the different tools and because WGS data is not necessary for fcircRNA detection.

For Acfs, reads were prepared using the recommended steps for paired-end sequencing data provided on the project’s GitHub page (<https://github.com/arthuryxt/acfs>).

**References**

Danecek,P. *et al.* (2021) Twelve years of SAMtools and BCFtools. *GigaScience*, **10**, giab008.

Liao,Y. *et al.* (2019) The R package Rsubread is easier, faster, cheaper and better for alignment and quantification of RNA sequencing reads. *Nucleic Acids Research*, **47**, e47.

Tate,J.G. *et al.* (2019) COSMIC: the Catalogue Of Somatic Mutations In Cancer. *Nucleic Acids Research*, **47**, D941–D947.

Wu,T.D. and Nacu,S. (2010) Fast and SNP-tolerant detection of complex variants and splicing in short reads. *Bioinformatics*, **26**, 873–881.

Zhang,J. *et al.* (2016) INTEGRATE: gene fusion discovery using whole genome and transcriptome data. *Genome Res.*, **26**, 108–118.

**Supplementary Figure 1. Junction annotation strategy used by INTEGRATE-Circ.** Simplified schematic of how INTEGRATE-Circ compares secondary junctions to the primary fusion junction in order to assign annotations.

**Supplementary Figure 2. GSNAP alignment of HCC1395 fusion reads reported by Fcirc.** A) RNGTT:RPF1 supporting reads are aligned to RPF1 and BUB1B by GSNAP and were therefore missed by INTEGRATE-Circ. B) Both NRL::DHRS4-AS1 supporting reads are spanning reads, but INTEGRATE-Circ requires encompassing read support for all fusions. C) ZNF37BP::CEP164P1 supporting reads reported by Fcirc are primarily mapped to an intergenic region by GSNAP and are therefore missed by INTEGRATE-Circ.

**Supplementary Figure 3. Quantification of fcircRNA expression in HCC1395 and HCC1395BL.** A-C) Relative expression of fcircRNA candidates 1-3 based on PrimeTime Probe qPCR. Expression values for each candidate are normalized to the expression found in HCC1395BL. *- In the case of Candidate 3, no expression was detected for HCC1395BL. For visualization pruposes, HCC1395BL expression was therefore set to a “hypothetical” Tm value of 36.


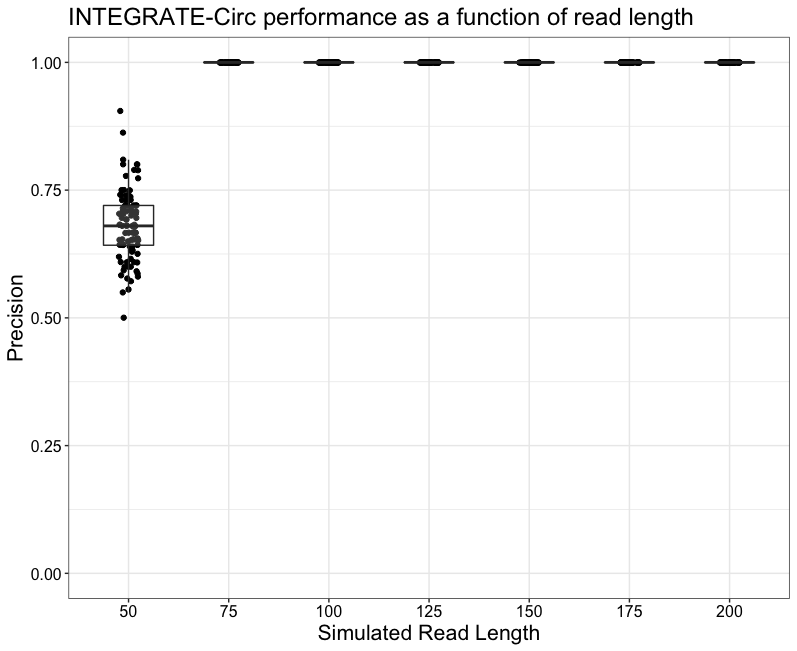


**Supplementary Figure 4.** INTEGRATE-Circ performance as a function of read length using simulation data
